# Using a Powered Bone Marrow Biopsy System Results in Shorter Procedures, Causes Less Residual Pain to Adult Patients, and Yields Larger Specimens

**DOI:** 10.1186/1746-1596-6-23

**Published:** 2011-03-23

**Authors:** James R Berenson, Ori Yellin, Brent Blumenstein, Deanna Bojanower, Jonathan Croopnick, David Aboulafia, Gargi Upadhyaya, Cathy Spadaccini

**Affiliations:** 1 Oncotherapeutics, Inc., 9201 W. Sunset Blvd, W. Hollywood, CA 90069, USA; 2 Institute for Myeloma and Bone Cancer Research, 9201 W. Sunset Blvd., Suite 300, W. Hollywood, CA 90069, USA; 3 Trial Architecture Consulting, 2240 Cathedral Ave NW, Washington, DC 20008, USA; 4 Northern Utah Associates, 4403 Harrison Blvd, Ste. 1685, Ogden, UT 84403, USA; 5 MetroWest Medical Center, 115 Lincoln St, Framingham, MA 01702, USA; 6 Virginia Mason Medical Center and the University of Washington, 1100 Ninth Ave, Seattle, WA 98101, USA; 7 Wilshire Oncology Medical Group, Inc., 210 S. Grand Ave, Suite 402, Glendora, CA 91741, USA; 8 Ameripath, South Texas, 301 N. Frio, San Antonio, TX 78207, USA

## Abstract

**Background:**

In recent years, a battery-powered bone marrow biopsy system was developed and cleared by the U.S. Food and Drug Administration to allow health care providers to access the bone marrow space quickly and efficiently. A multicenter randomized clinical trial was designed for adult patients to determine if the powered device had advantages over traditional manually-inserted needles in regard to length of procedure, patient pain, complications, user satisfaction, and pathological analysis of the specimens.

**Methods:**

Adult patients requiring marrow sampling procedures were randomized for a Manual or Powered device. Visual Analog Scale (VAS) pain scores were captured immediately following the procedure and 1 and 7 days later. Procedure time was measured and core specimens were submitted to pathology for grading.

**Results:**

Ten sites enrolled 102 patients into the study (Powered, n = 52; Manual, n = 50). Mean VAS scores for overall procedural pain were not significantly different between the arms (3.8 ± 2.8 for Powered, 3.5 ± 2.3 for Manual [p = 0.623]). A day later, more patients who underwent the Powered procedure were pain-free (67%) than those patients in the Manual group (33%; p = 0.003). One week later, there was no difference (83% for Powered patients; 76% for Manual patients.) Mean procedure time was 102.1 ± 86.4 seconds for the Powered group and 203.1 ± 149.5 seconds for the Manual group (p < 0.001). Pathology assessment was similar in specimen quality, but there was a significant difference in the specimen volume between the devices (Powered: 36.8 ± 21.2 mm^3^; Manual: 20.4 ± 9.0 mm^3^; p = 0.039). Two non-serious complications were experienced during Powered procedures (4%); but none during Manual procedures (p = 0.495).

**Conclusions:**

The results of this first trial provide evidence that the Powered device delivers larger-volume bone marrow specimens for pathology evaluation. In addition, bone marrow specimens were secured more rapidly and subjects experienced less intermediate term pain when the Powered device was employed. Further study is needed to determine if clinicians more experienced with the Powered device will be able to use it in a manner that significantly reduces needle insertion pain; and to compare a larger sample of pathology specimens obtained using the Powered device to those obtained using traditional manual biopsy needles.

## Background

Bone marrow evaluation is often essential to determine the efficacy of treatment in hematological disorders and to monitor the recovery process in patients undergoing bone marrow transplantation or marrow-ablative chemotherapy [1,2]. It is also an essential component of the staging process for patients newly diagnosed with lymphoproliferative diseases and certain non-hematopoietic malignancies. Bone marrow examination is instrumental in determining the extent of marrow damage among patients exposed to radiation, drugs, chemicals, and other myelotoxic agents [3].

Since the introduction of the Jamshidi needle in 1971, there has been no substantial advancement in marrow sampling technology [4]. Biopsy procedures facilitated by drill-powered needles have been attempted before, but the devices were prototypes and were never commercially available [5,6]. In recent years, a battery-powered bone marrow biopsy system was developed and cleared by the U.S. Food and Drug Administration to allow health care providers to access the bone marrow space quickly and efficiently. The OnControl powered device (Vidacare Corporation, Shavano Park, TX) was introduced for bone marrow aspirates in 2007. An improved design featuring an integrated sterile tray needed for core biopsies was introduced for this study in 2009 and made available for commercial sales in 2010. The device utilizes a battery-powered drill to insert the bone marrow needle into the iliac bone of adult patients with minimal operator exertion. An evaluation of the first generation device by Cohen and Gore indicated that the device was safe, as well as faster and easier to use than the traditional manual procedure [3]. The current generation of the device was also assessed in a non-controlled evaluation with similar results [7].

Patients with hematological disorders may require one or more bone marrow sampling procedures during their treatment process. Patients with cancer often suffer with more procedural pain than those with other disorders [8]. However, there are relatively few studies in the recent clinical literature that address the issue of pain management during the bone marrow sampling procedure. In a 2004 study of 263 patients undergoing bone marrow procedures, Kuball *et al *found that the duration of the procedure (typically 7 minutes) was the sole independent factor for patients' pain intensity [9]. Antmen *et al *reported VAS pain scores ranging from 1.8 to 3.5, in an 80-patient study of children receiving bone marrow procedures under sedation, depending on the agent used for sedation [10]. In a 2007 study by von Gunten and Soskinks, technicians rated their perception of patient pain during the bone marrow procedure. The investigators found that the perceived pain scores correlated with the difficulty of performing the procedure [11]. The results from both of these studies suggest that procedural difficulties cause longer procedures, resulting in increased pain for the patient. In a 2009 study comparing biopsy pain when using standard Lidocaine and buffered Lidocaine, Ruegg *et al *reported mean biopsy needle insertion pain to be 38.5 (on a scale of 0-100, with higher scores indicating more pain) when patients were anesthetized with buffered Lidocaine. In a study involving 235 patients conducted by Liden *et al *in 2009, 70% of the patients reported pain during and after bone marrow biopsy procedures. At 1, 3, 6 and 7 days following the bone marrow procedure, pain was present in 137 (64%), 90 (42%), 43 (20%) and 25 (12%) patients, respectively [12].

Some providers elect to use conscious sedation during the bone marrow procedures in an attempt to mitigate procedural pain and discomfort; but this takes longer and may expose the patient to additional physical risks, result in increased liability for the provider, and require increased patient monitoring during and after the procedure [13]. In a study of 138 patients evaluating the effects of lorazepam on pain during bone marrow procedures, Park *et al *reported mean VAS scores during the bone marrow procedures as 6.0 ± 2.5 for lorazepam vs. 6.2 ± 2.3 for placebo [14]. Each of those patients also received a local injection of 1% Lidocaine. In 2010, Degen *et al *conducted survey of 412 patients that assessed pain during the bone marrow sampling procedure. In this study, 336 patients received opioids or benzodiazepines prior to the procedure. Medication-related complications occurred in 32.7% of patients, with the most frequent complications being tiredness (22.6%), dizziness (5.7%), and nausea (4.5%) [15].

Until now no data has been available that compares patient outcomes between Powered and Manual bone marrow sampling devices. We report the results of this first randomized controlled study conducted comparing the Powered bone marrow sampling system to traditional manual biopsy needles in regard to length of procedure, patient pain, complications, user satisfaction, and pathological analysis of the specimens.

## Methods

**Figure 1:**
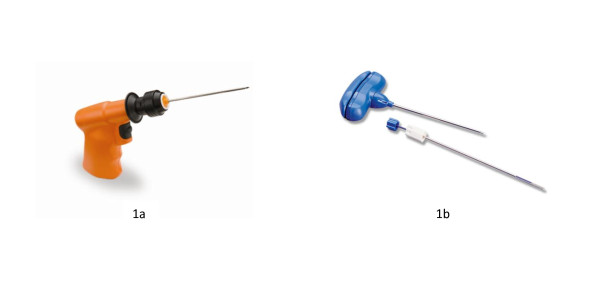
**Figures 1a and 1b**. Power driver and biopsy needle components of the OnControl powered bone marrow sampling system, and typical Jamshidi-type manual bone marrow biopsy needle.

This multi-center randomized, controlled trial was approved by Western Institutional Review Board; and the study was conducted in community-based cancer clinics in accordance with ethical standards described in the 1964 Declaration of Helsinki. Patients requiring a bone marrow biopsy provided written informed consent and were randomized to receive the procedure using either a Powered or Manual bone marrow needle. The Powered device was the OnControl Bone Marrow Biopsy System (Vidacare Corporation, Shavano Park, TX), an FDA-cleared device consisting of a powered driver and biopsy needle set. The battery-powered driver resembles a small hand-held drill and drives a single lumen needle set into the medullary cavity of the adult iliac crest. The needle set consists of two parts: an outer cannula, 11 gauge × 4 inches (102 mm) long; and a bevel-tip inner stylet--used to penetrate the cortex of the bone (Figure 1a). The specific Manual device varied across study sites but was typically a Jamshidi-type bone marrow biopsy needle, 11 gauge × 4 inch, which has a two-piece T-handle design, a trocar-tapered stylet point and a triple crown cannula tip (Figure 1b).

Operators were skilled in the use of the Manual devices but had limited experience using the Powered device. Each operator was required to use the Powered device on 3 to 5 non-study patients before use on study patients. Study patients at each site were randomized to receive a bone marrow biopsy procedure with either the Powered device or a Manual device. Targeting the posterior iliac crest, biopsy procedures were performed in accordance with local policy and device directions for use. Procedure time was measured as time in seconds from contact of the needle with the skin to needle removal. Patients assessed pain at the end of the procedure using a Visual Analog Scale (VAS), with scores ranging from 0 to 10; higher scores indicating greater pain. Pain was also assessed at one day and 7 days after the biopsy procedure. Device-related complications and adverse events were recorded. Operator and patient satisfaction with the biopsy device were assessed on a scale of 0 to 10, with higher scores indicating higher satisfaction.

One biopsy specimen acquired using each device type from each study site was submitted to a central pathologist for quantitative and qualitative analysis. Core specimen measurements (length and width) were made after bone marrow fixation and processing. The general quality of the bone biopsy was examined after processing. After hematoxylin and eosin staining, microscopic slides were assessed for fragmentation, crush artifact, hemorrhage, trabecular distortion, cell viability and other artifacts, and scored for quality on a scale of 1 to 4, with 4 indicating excellent.

The quality of the medullary component of the biopsy was assessed separately to determine if sufficient medullary marrow was present to evaluate the cells in the marrow and graded on a scale of 0 to 4, with 4 indicating excellent. The overall quality of the biopsy was assessed as to whether the sample was adequate, suboptimal but helpful, or inadequate for a diagnosis.

After the interim analysis, the protocol was amended to include recording the patient's pain level immediately following bone cortical penetration by the biopsy needle. Patient satisfaction with the device was also assessed on a scale of 0 to 10, with higher scores indicating higher satisfaction. These additional data points were recorded only after obtaining IRB approval for the protocol revision. At the time of the protocol revision, only 28 of the 102 enrolled patients remained for accrual into that portion of the study.

Patients' demographics were evaluated to determine balance across the two study arms in terms of age, sex, number of prior bone marrow aspirations, and amount of pain on previous bone marrow aspiration. Statistical tests were conducted using SAS 9.2 (SAS Institute Inc., Cary, NC) and IBM SPSS Statistics 19.0 (SPSS, Inc. Chicago, IL). Continuous parameters were summarized and compared between groups using a 2-sample *t*-test. Categorical parameters were summarized as proportions and compared using Fisher's Exact test. A priori significance level was set at 0.05.

## Results

Thirteen device operators from 10 sites participated in the study. All patients who completed the randomization process (102 total; Powered, n = 52; Manual, n = 50) received the bone marrow sampling procedure. The mean age of participating patients was 66.3 ± 14.1 years and 56% were male. The mean height of the patients was 169.0 ± 10.2 cm and the mean weight was 76.3 ± 16.7 kg. There were no significant differences in the means for these variables across the two arms of the study. Forty-four percent of the patients had hematological disorders. See Table 1. This was the first bone marrow biopsy for 69% of Powered and 56% of Manual patients.

**Table 1:** Patient demographics by device type

Variable	Manual	Powered	p-value
Male/female (%)	60.0/40.0	51.9/48.1	0.432

Mean age (years)	66.4 ± 13.4	66.2 ± 14.7	0.952

Mean height (cm)	169.9 ± 10.8	168.0 ± 9.5	0.355

Mean weight (kg)	78.1 ± 16.5	74.7 ± 16.9	0.312

Mean body mass index	26.9 ± 4.4	26.4 ± 5.4	0.588

Frequency of disease categories			0.135
Lymphoma	12	14	
Leukemia	13	12	
Multiple myeloma	10	8	
Hematological	0	6	
Leukemia/lymphoma	2	0	
Other	13	12	

Mean VAS scores for overall procedural pain were not significantly different between the arms (3.8 ± 2.8 for Powered and 3.5 ± 2.3 for Manual [*p *= 0.623]). Assessment of needle insertion pain was done on the last 28 patients enrolled in the study. VAS pain scores were not different between the arms (3.1 ± 3.1 for Powered [n = 14] and 3.2 ± 2.9 for Manual [n = 14]). One day after the procedure, more patients who underwent the Powered procedure were pain-free (67%) than those patients in the Manual group (33%; *p *= 0.003). One week following the procedure, there was no difference in the proportion of patients who were pain-free (83% for Powered patients; 76% for Manual patients). Notably, mean procedure time was shorter for the Powered group (102.1 ± 86.4 seconds) than for the Manual group (203.1 ± 149.5 seconds; *p *< 0.001). Biopsy core acquisition success rate was similar between the arms (Powered: 90.4%; Manual: 98.0%; *p *= 0.205). Assessment by pathology showed equivalence in core specimen quality parameters and length and width, but the specimen volume was larger in the Powered group (36.8 mm^3 ^± 21.2) than the Manual group (20.4 mm^3 ^± 9.0; *p *= 0.039). There was no difference between the two devices for operator satisfaction, nor for patient satisfaction. There were two non-serious complications for the Powered (3.8%). In one case, the patient's skin became wrapped around the shaft of the rotating biopsy needle; and in the other case, the clinician's glove became wrapped for a very brief time around the rotating biopsy needle. There were no complications for the Manual procedure (*p *= 0.495). See Table 2.

**Table 2:** Results by device type

Device Efficacy	Manual	Powered	p-value
Able to acquire biopsy core specimen	98.0%	90.4%	0.205

Mean number attempts for core acquisition	1.5 ± 0.8	1.2 ± 0.5	0.112

Mean time core acquisition (seconds)	203.1 ± 149.5	102.1 ± 85.4	0.000*

**Patient Pain Scores**	**Manual**	**Powered**	**p-value**

Mean VAS needle insertion (0-10)	3.2 ± 2.9	3.1 ± 3.1	0.900

Mean VAS overall (0-10)	3.5 ± 2.3	3.8 ± 2.8	0.623

Pain-free patients 24 hours post procedure	33.3%	66.7%	0.003*

Pain-free patients 7 days post procedure	76.1%	82.6%	0.607

**Satisfaction & Complications**	**Manual**	**Powered**	**p-value**

Operator satisfaction (0-10)	7.8 ± 2.1	8.1 ± 1.7	0.488

Patient satisfaction (0-10)	9.3 ± 1.6	9.4 ± 1.2	0.778

Device related complications	0%	3.8%	0.495

**Core Biopsy Pathology Assessment**	**Manual**	**Powered**	**p-value**

Mean length (mm)	11.0 ± 5.5	13.3 ± 6.6	0.368

Mean width (mm)	1.3 ± 0.5	1.7 ± 0.5	0.123

Mean volume (mm^3^)	20.4 ± 9.0	36.8 ± 21.2	0.039*

General quality excellent/good	80.0%	66.7%	0.628

Medullary quality excellent/good	70.0%	55.6%	0.650

Overall quality rated adequate	70.0%	77.8%	1.000

*denotes statistical significance

## Discussion

We propose that a major advantage of the Powered system is that it allows clinicians to more quickly complete the bone marrow sampling procedure. In our study, the mean time from needle to skin contact to removal of the needle using the Powered system was approximately half the time required using the Manual device 102.1 ± 86.4 seconds vs. 203.1 ± 149.5 seconds, respectively). Patients undergoing bone marrow biopsy procedures most likely expect some level of pain. Most patients are willing to undergo the procedure and a reasonable level of pain, providing the procedure time is relatively short. Assuming the validity of Kuball *et al*'s finding that procedure time has the greatest impact on pain, use of the Powered system could have a positive effect on many patients' perception of the bone marrow procedure in general.

Another important finding from our study is that whereas patients generally expect pain during any invasive procedure, they may not expect or tolerate persistent residual pain afterwards. In our study, 67% of the patients who received the bone marrow biopsy procedure using the Powered device were pain-free within 24 hours compared to only 33% of patients who received the procedure with the Manual device, a statistically and clinically significant finding. In the Liden *et al *study using the Manual procedure, only 36% of patients were without pain after 24 hours, very similar to the results in the Manual group in our study [12].

As expected, there were few complications in our study with either the Powered or Manual bone marrow biopsy procedures. The two observed complications occurred with a single operator's use of the Powered device. In one case, a small portion of the patient's skin wrapped around the rotating needle. This could have been avoided by holding the skin tautly at the needle insertion site. In the other case, the operator's latex glove became wrapped around the rotating needle, which could have been avoided by not placing the gloved finger too close to the rotating needle. It is recognized that these types of complications are potentially serious and not likely to occur with manual devices. But it is felt that complications of this type can be avoided by emphasizing this potential problem and methods to avoid the problem during operator training. Moreover, considering that both these problems were experienced by only one of 13 operators participating in the study, their prevalence is not felt to be wide-spread.

Clinicians attempt to conduct bone marrow sampling procedures quickly and safely with as little pain and discomfort to the patient as possible; however, the ultimate goal of the procedure is to acquire a bone marrow specimen that is adequate for diagnosis by pathologists. Adequate specimens need to be of adequate size and free of crush trabecular distortion and other artifact. Inadequate specimens can diminish the clinician's ability to make an accurate diagnosis and/or assessment of the patient's clinical status. Bishop *et al *found only 42% of bone marrow biopsy specimens were adequate for accurate diagnosis in a study involving 767 patients [16]. In the current study, there were no differences between the two procedure types in specimen quality or length; however, use of the Powered device resulted in bone marrow core specimens that had 80% more volume than specimens obtained with the Manual device. Although length is generally the criteria used for determining the ideal size of a bone marrow core specimen, specimen volume may be a more relevant indicator of the amount of tissue available for analysis. Larger tissue volume of the specimen may increase the ability of the pathologist to conduct a thorough analysis and identify focal lesions.

The cost of using the Powered device must be considered in decisions regarding its use in the clinic. While the powered driver can be used up to 500 times, the needle and other components are for single use and part of a tray that generally costs $60 to $70 more than the Manual device. Although needle-for-needle, the cost of the Powered system is higher, the total cost for the disposable needle tray is covered by insurance reimbursement under most plans. While the seemingly high cost of the Powered device might negatively influence a decision to adopt its use, the concerns of most clinicians and patients are not simply the costs of the material and equipment required to do the procedure but the costs of these items together with their quality and patient outcome. Our study and other recent studies [17,18], suggest that the Powered system enables clinicians to consistently obtain biopsy specimens of superior size and quality than those obtained using the Manual needles. In many cases, the lower cost of the Manual device may be offset by the necessity to repeat the procedure--requiring more materials and clinician time. Regardless of costs, we feel using the Powered system adds value to the process in terms of speed, efficiency, and patient comfort.

There were several limitations in this study. As with any new device, there is a learning curve that must be negotiated before operators can gain proficiency. While our study protocol stipulated that the operator complete 3 to 5 Powered device uses in non-study subjects before performing the procedure on study patients, in hindsight perhaps those numbers should have been higher. Another limitation was initially capturing patient pain scores as an "overall" event that included the aspiration portion of the bone marrow sampling procedure. Generally, bone marrow aspiration is painful regardless of the needle-type used to withdraw the liquid bone marrow specimen. This extreme pain during the aspiration phase may obscure any differentiation in pain levels between the two device types during the less painful phases of the procedure. This phenomenon was realized after the interim analysis. By the time the study protocol could be revised to include recording needle insertion pain, patient accrual was 72% complete. Thus, we were unfortunately able to collect very little data specifically on needle insertion pain. A final limitation was that relatively few core specimens were available for pathology analysis. Our initial plan was to conduct quality analysis for each specimen at a central laboratory, but some investigators were reluctant to send specimens to the central laboratory following analysis at their local laboratory. The compromise was that two specimens from each center were analyzed, one for each device type.

## Conclusions

The results of this first multicenter randomized controlled trial evaluating the Powered bone marrow biopsy system suggest that use of the Powered bone marrow biopsy device may deliver larger-volume bone marrow core specimens for analysis, markedly shorten the procedure time, and reduce intermediate-term pain--important considerations for both the medical provider and the patient when determining how best to obtain bone marrow specimens. Further study is needed to determine if clinicians more experienced with the Powered device will be able to use it in a manner that significantly reduces needle insertion pain; and to compare a larger sample of pathology specimens obtained using the Powered device to those obtained using traditional manual biopsy needles.

## Competing interests

All authors, or their respective organizations, received research grant funds from the sponsor of the study, Vidacare Corporation, for their participation in the study.

## Authors' contributions

JRB and OY participated in the design of the study, collected clinical data, and helped draft the manuscript. BB participated in the design of the study and performed the statistical analysis. DB, JC, DA, GU collected clinical data and helped draft the manuscript. CS participated in the design of the pathology portion of the study and helped draft the manuscript. All authors read and approved the final manuscript.
